# The Effects of Group Fitness Programs Zumba and MoFit on Body Composition Parameters in Women

**DOI:** 10.3390/life15081225

**Published:** 2025-08-03

**Authors:** Armin Zećirović, Dejan Ćeremidžić, Aleksandar Joksimović, Tatjana Ćeremidžić, Dina Joksimović, Nikola Aksović, Lazar Toskić, Cristian-Corneliu Dragoi, Vasile Cătălin Ciocan, Anghel Mihaela, Tatiana Dobrescu, Daniel-Lucian Dobreci

**Affiliations:** 1 Faculty of Physical Education and Sports, University of East Sarajevo, 71126 Lukavica, Bosnia and Herzegovina; armin.zecirovic@gmail.com (A.Z.); dorapet@teol.net (D.Ć.); tceremidzic@yahoo.com (T.Ć.); 2 Evergrande Football School, Guangzhou Football Club, Qingyuan 511500, China; 3 Institute of Applied Technology, Abu Dhabi 20300, United Arab Emirates; alex.joksimovic@actvet.gov.ae (A.J.); dina.joksimovic@actvet.gov.ae (D.J.); 4 Faculty of Sport and Physical Education, University of Priština-Kosovska Mitrovica, 38218 Leposavić, Serbia; kokir87np@gmail.com; 5 Faculty of Sport, University “Union–Nikola Tesla”, 11070 Belgrade, Serbia; 6 Faculty of Movement, Sport and Health Sciences, “Vasile Alecsandri” University of Bacau, 600115 Bacau, Romania; cristi_dragoi@ub.ro (C.-C.D.); anghel.mihaela@ub.ro (A.M.); tatiana.dobrescu@ub.ro (T.D.); dobreci.lucian@ub.ro (D.-L.D.)

**Keywords:** group exercise, women, body fat, muscle mass, total body water, minerals

## Abstract

(1) Background: Physical inactivity is a major public health concern in modern society. Group fitness programs are widely used to promote physical activity, combining choreographed movements with various dance steps and music. This study aimed to examine the effects of Zumba and MoFit group fitness programs on body composition parameters in women. (2) Methods: The study included 98 female participants (Mean age = 27.8 ± 2.9 years), divided into three groups: E1 (n = 33), which followed the experimental Zumba program; E2 (n = 31), which followed the experimental MoFit program; and a control group (n = 34), which continued with their usual daily activities for 10 weeks. Body composition was assessed using 14 variables measured with the InBody 270 analyser. Statistical analyses included paired *t*-tests, MANCOVA, and ANCOVA. (3) Results: The findings confirmed the positive effects of both group fitness programs on most body composition parameters in women (*p* < 0.001). However, Bonferroni post hoc test results indicated that the Zumba program led to significantly greater improvements in most body composition variables compared to the MoFit program. (4) Conclusions: Both Zumba and MoFit programs were effective in reducing body fat, increasing muscle mass, total body water, and mineral content, whereas the control group did not achieve positive changes.

## 1. Introduction

Physical inactivity is one of the most significant health concerns in modern society, contributing to the development of chronic diseases, various disorders, and premature mortality, with this trend increasing each year [1,2]. Regular and appropriately structured physical activity has a preventive effect, reducing the risk of cardiovascular diseases, diabetes, osteoporosis, stress, anxiety, depression, and other prevalent health conditions [3,4,5,6]. In discussions on physical inactivity, it is essential to consider related factors such as obesity and alterations in body composition, primarily characterised by increased adipose tissue and decreased muscle mass, both of which play a crucial role in overall health [7,8].

Group fitness programs are a fundamental form of physical activity that incorporates choreography with various dance steps and music, making them one of the most popular exercise modalities, particularly among women [9,10]. These programs are designed to enhance physical appearance and improve overall health, including cardiorespiratory fitness, body composition, flexibility, and muscular strength [11,12]. Such programs vary in content, biomechanical parameters, purpose, and the use of equipment and props. Women often opt for group fitness programs due to the diversity of available exercise options, regardless of age or fitness level [13]. Additionally, exercising with music and choreography, combined with the use of various props under the supervision of an instructor, creates an enjoyable and engaging experience [14].

It is important to note that group fitness programs are continuously evolving worldwide, incorporating new elements and undergoing modifications to enhance effectiveness [15]. While some programs differ in name, this does not necessarily affect the originality or core structure of the program itself. However, such distinctions are often employed for marketing purposes to attract a larger number of participants [16]. Zumba is a globally recognised and popular workout that blends Latin rhythms with various dance movements, entertainment, and fitness [15,17]. Meanwhile, emerging fitness programs such as MoFit (Movement Fitness) integrate elements from multiple disciplines, including strength training, cardiovascular conditioning, functional movement, and stretching. MoFit is a contemporary, innovative group fitness program designed to integrate various training elements and support comprehensive physical development and fitness. It is suitable for all levels, from beginners to advanced practitioners, with workouts that can be individually tailored to each participant’s goals, whether those are weight loss, improved conditioning, or general health. The program places special emphasis on functional movements that contribute to improved body composition, strength, and coordination, thereby reducing the risk of injury in daily life. Stretching and mobility exercises are also integral parts of the program, as they increase flexibility, alleviate muscle tension, and promote faster recovery after training. It should be emphasised that the MoFit program was developed at an institutional level; however, a formal validation has not yet been published in the scientific literature.

Numerous studies have explored the effects of group fitness programs, making this a highly active field of research. Baranco Ruiz et al. [18] found that choreographed group fitness activities, such as Zumba and Zumba combined with bodyweight exercises, positively impact body composition, cardiovascular health, and metabolic function in physically inactive women. However, their study found no significant differences between the two experimental groups. Similarly, Bjelica [19] demonstrated that a 12-week Zumba and fitness program induced positive changes in body composition, with Zumba proving more effective than general fitness training. In contrast, Chavarrias et al. [20] found that a fitness program centred on indoor cycling (IC), whether alone or combined with Zumba or Body Pump, significantly reduced blood pressure and improved body composition and physical fitness in women. Notably, the IC program was the most effective in reducing body weight and fat mass. Additionally, Ljubojević et al. [21] suggested that an eight-week Zumba fitness program positively influences respiratory function and body composition in healthy, inactive women. However, contradictory results were reported by Delextat et al. [22], who found that an eight-week Zumba program did not lead to significant improvements in body composition among healthy women. These discrepancies underscore the need for further research to fully elucidate the effects of group fitness programs on women’s body composition.

This is the first study to provide a comprehensive investigation of body composition changes following the implementation of these specific fitness programs. Zumba is already an established international fitness program, while MoFit is an institutionally developed program. Therefore, comparing these two approaches provides insight into the effectiveness of different methodologies in group fitness training. The primary aim of this study was to examine the effects of Zumba and MoFit group fitness programs on body composition parameters in women. Accordingly, we hypothesised that Zumba and MoFit fitness programs would exert significant but distinct effects on women’s body composition.

## 2. Materials and Methods

In this study, a longitudinal research design was employed. Baseline body composition measurements were taken immediately before the start of the experimental program. Following the completion of the 10-week Zumba and MoFit experimental programs, final assessments were conducted for both the experimental and control groups.

### 2.1. Sample of Participants

The sample consisted of 98 female participants, who were divided into three groups:

E1—First experimental group (n = 33), with an average age (26.5 ± 5.6 y), body height (168.5 ± 7.6 cm), body mass (67.4 ± 12.9 kg), and body mass index (23.8 ± 4.9 kg/m^2^). This group participated in the Zumba experimental program.

E2—Second experimental group (n = 31), with an average age (31.2 ± 9.1 y), body height (167.6 ± 6.7 cm), body mass (65.7 ± 13.8 kg), and body mass index (23.3 ± 4.5 kg/m^2^). This group participated in the MoFit experimental program.

C—Control group (n = 34), with an average age (25.9 ± 7.0 y), body height (169.0 ± 5.7 cm), body mass (63.8 ± 11.7 kg), and body mass index (22.2 ± 3.3 kg/m^2^). This group did not engage in any form of organised exercise but continued with their usual daily activities. Inclusion and exclusion criteria were established to determine eligibility.

Inclusion criteria:

- Healthy female individuals without acute or chronic diseases or injuries;
- Not engaged in any other form of organised exercise or participating in other structured physical or sports activities;
- Voluntarily agreed to participate in the experiment and attend group fitness training sessions regularly for a duration of 10 weeks;
- Not pregnant and not in the postpartum recovery period;
- Have not used medications or supplements that may affect metabolism.

Exclusion criteria:

- Women who were involved in another organised form of exercise;
- Women with cardiovascular and respiratory diseases;
- Women in the process of recovery from any acute or chronic illnesses;
- Women undergoing rehabilitation from injuries;
- Women who use medications or supplements that may affect the results;
- Women with uncontrolled menstrual irregularities, metabolic or hormonal disorders.

Since all participants voluntarily gave their written consent to participate in the study, they were allowed to withdraw from the experimental programs at any time during the program’s duration. The research was carried out in accordance with the conditions of the Declaration of Helsinki, and the study was approved by the ethical board of the Faculty of Sport and Physical Education (IRB: 03-779/1).

### 2.2. Measurement Procedures

The measurement of body composition was performed using InBody 270 (Biospace Co., Ltd., Seoul, Republic of Korea). The measurement procedures were conducted following the manufacturer’s recommendations and similar previous studies [23,24,25]:

- Measurements were taken in the morning between 8:00 and 10:00 a.m.;
- Participants were asked to abstain from large meals after 9 p.m. the day before testing;
- Participants were asked to abstain from eating and drinking prior to testing on the measuring day;
- Participants were asked to refrain from extreme physical exertion 24 h prior to measuring, and the last training should have been performed at least 12 h prior to measuring;
- Participants were asked to abstain from consuming any alcoholic drinks 48 h before measuring;
- Participants were asked to urinate and defecate at least 30 min prior to measuring,
- Participants were in the standing position at least 5 min prior to measuring due to normal fluid distribution in the body;
- Measuring was taken in the standing position, as was suggested by the manufacturer (hands aside, placed 15 cm laterally from the body).

The following variables were used to assess body composition, as shown in Table 1.

It should be noted that the participants’ height and body mass were measured, but the obtained data were not subjected to statistical analysis; they served only as an identification of the anthropometric measures of the participants on whom the research was conducted. Additionally, based on the values of height and body mass, the body mass index (BMI) was also calculated. It is important to note that the InBody 270 does not directly measure bone density but rather estimates mineral content based on indirect parameters. Furthermore, although the InBody 270 displays the values of the total body water (TBW) variable in kilograms, the authors presented the results as percentages to facilitate easier understanding and interpretation.

### 2.3. Zumba and MoFit Interventions

The first experimental group followed the Zumba program, while the second experimental group followed the MoFit program. Zumba and MoFit classes were led by specialised and licensed fitness instructors. Both experimental programs were conducted for 10 weeks [9,17], with three training sessions per week, at the “MVP ACTIVE” Fitness Centre in Novi Pazar, Serbia. The intensity of each modality was controlled by monitoring heart rate. Throughout the entire exercise session, heart rate was monitored during the warm-up, main, and cool-down phases for both groups. Participants were educated on the importance of maintaining heart rate within target zones that align with their fitness objectives. In addition, instructors visually observed participants throughout each session, carefully monitoring for signs of fatigue, loss of form, laboured breathing, or excessive sweating. Based on these observations, instructors were able to adjust the pace and complexity of movements in real time. Table 2 presents the basic information related to the execution of the experimental programs.

The Zumba sessions lasted between 65 and 70 min and were conducted by licensed instructors. Training intensity ranged from 70% to 85% of the maximum heart rate (HRmax). The program consisted of three segments: an introductory phase (7–10 min) incorporating basic dance steps (Bollywood, Kuduro, Dancehall, Salsa, etc.), a main phase featuring musical choreographies from various dance styles (Salsa, Merengue, Cumbia, Reggaeton, Pasodoble, and Show Dance), and a concluding phase (5–8 min) focused on stretching and muscle relaxation. The program was designed to enhance body composition in women [15,19,20,21].

The experimental MoFit program sessions lasted between 55 and 60 min, with an intensity range of 60% to 75% of HRmax. The program emphasised targeted muscle activation and body composition transformation. The introductory phase (7–10 min) included warm-up exercises such as walking, running, and basic shaping movements. The main phase consisted of structured sets of exercises targeting different muscle groups, including the legs (squats, lunges, and calf raises), back (Y, T, and I raises and extensions), arms (dips and bicep curls with weights), abdominal muscles, and shoulders (crunches, planks, and wood chop). Some sessions followed a circuit-style format (Full Body program).

The final phase (5–8 min) incorporated stretching and muscle relaxation exercises set to music. The intensity level was moderate, with exercises performed using bodyweight or light resistance (resistance bands, 0.2–1 kg weights, and 2–3 kg medicine balls).

Participants in the control group did not engage in any structured exercise program and continued with their usual daily activities, classifying them as inactive women.

### 2.4. Statistical Analysis

Data processing employed the statistical program SPSS (v25.0, SPSS Inc., Chicago, IL, USA). For all results obtained through testing, basic descriptive parameters were calculated: mean (Mean) and standard deviation (SD).

To determine the differences between the initial and final measurements, a paired *t*-test was applied, along with the calculation of effect size (Cohen’s d) within each group. Cohen’s guidelines for interpreting the effect size are as follows: 0.01—small impact, 0.06—moderate impact, and 0.14—large impact [26]. Preliminary analyses determined that the assumptions of normal distribution (Kolmogorov–Smirnov test), linearity, homogeneity of variances, and homogeneity of regression slopes were not violated.

The effects of the experimental program were assessed using multivariate analysis of covariance (MANCOVA) and univariate analysis of covariance (ANCOVA), with the calculation of effect size (Partial Eta Squared). Differences were tested using the F-test. The results from the initial measurement were used as a covariate to control for baseline differences, in order to further ensure the accuracy and validity of the findings and to more precisely present the effects of the implemented fitness programs.

Confidence intervals (95% CIs) were calculated for the key outcomes to provide an estimate of the precision of the results. The sample size (total n = 98) was considered sufficient to perform the applied statistical tests and to detect clinically relevant differences based on commonly accepted guidelines for these analyses [26]. The level of significance (*p*) was set at *p* < 0.05 level.

## 3. Results

### 3.1. Differences in Body Composition Between Initial and Final Measurements of Experimental and Control Groups

Table 3 presents the results of the *t*-test used to determine the differences between the initial and final measurements in the applied body composition variables for the Zumba experimental group. Upon reviewing the results, it can be concluded that statistically significant differences were found between the initial and final measurements for all variables at a significance level of (*p* = 0.000, on average). It is important to note that, for all measured variables, numerical differences were observed in favour of better results in the final measurement compared to the initial one.

Based on the values of Cohen’s guidelines for interpreting the results, it can be concluded that the experimental Zumba program had a large positive impact on the transformation of body composition in all variables: LLW kg (0.83), TMW kg (0.80), RLW kg (0.79), RHW kg (0.73), LHW kg (0.66), TBF % (0.58), RHF % (0.55), LHF % (0.55), RLF % (0.55), TBW % (0.55), TTW kg (0.54), LLF % (0.39), TRF % (0.25), and MIN kg (0.24).

In Table 4, the results of the *t*-test for determining differences between the initial and final measurements in the applied variables for assessing body composition in the MoFit experimental group are presented. A review of the obtained results shows that significant differences (*p* = 0.000, on average) were observed between the initial and final measurements in the following variables: TBF % (*p* = 0.000), RHF % (*p* = 0.001), LHF % (*p* = 0.000), TRF % (*p* = 0.000), RLF % (*p* = 0.000), LLF % (*p* = 0.000), TMW kg (*p* = 0.000), RLW kg (*p* = 0.000), LLW kg (*p* = 0.000), TBW % (*p* = 0.000), and MIN kg (*p* = 0.001). For the variable TTW kg (*p* = 0.041), the results indicate differences at the level of significance (*p* < 0.05), while no significant differences were observed for the other variables between the initial and final measurements (RHW %, LHW %, *p* = 0.573, on average). Certainly, it should be emphasised that, for all measured variables, numerical differences were observed in favour of better results at the final measurement compared to the initial measurement, except for the variable RHW kg, where the results remained the same.

Based on the values of Cohen’s guidelines for interpreting the results, it can be concluded that the MoFit experimental program had a large positive impact on the transformation of body composition in the following variables: TMW kg (0.89), LHF % (0.80), RLW kg (0.77), LLW kg (0.75), TBF % (0.58), RLF % (0.58), TBW % (0.55), TRF % (0.53), LLF % (0.46), RHF % (0.30), and MIN kg (0.29). For the variable TTW kg (0.13), a moderate impact was observed, while for the variables LHW kg (0.03) and RHW kg (0.01), a small impact was noted.

Table 5 presents the results of the *t*-test for determining the differences between the initial and final measurements in the applied variables for body composition assessment in the control group. Reviewing the obtained results, it can be stated that there were significant differences (*p* = 0.001, on average) between the initial and final measurements for the following variables: TBF % (*p* = 0.001), TRF % (*p* = 0.000), and TBW % (*p* = 0.002). Significant differences (*p* = 0.022, on average) were also found for the following variables: RHF % (*p* = 0.017), LHF % (*p* = 0.025), RLF % (*p* = 0.019), LLF % (*p* = 0.031), and TMW kg (*p* = 0.021). However, for the other variables, no significant differences were observed between the initial and final measurements (*p* = 0.406, on average). Certainly, it should be emphasised that, for most of the measured variables (TBF %, RHF %, LHF %, TRF %, RLF %, LLF %, and TBW %), numerical differences were observed in favour of poorer results in the final measurement compared to the initial measurement. However, for the variables (TMW kg, RHW kg, LHW kg, TTW kg, and LLW kg), numerical differences were noted in favour of better results in the final measurement compared to the initial measurement. For the variables RLW kg and MIN kg, the results remained unchanged.

**Figure 1:**
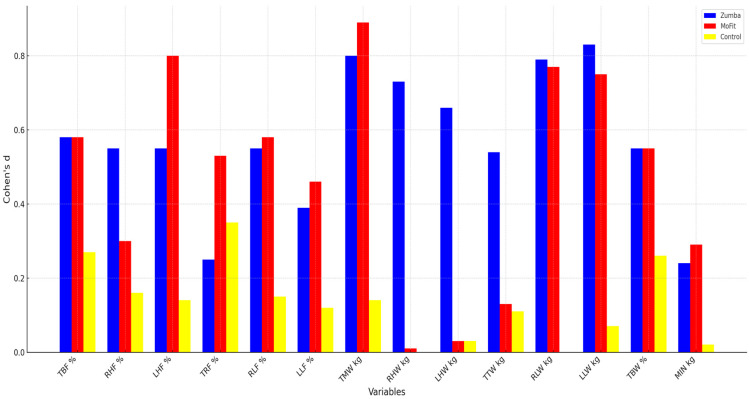
The Cohen’s d values for the body composition variables.

Based on the values of Cohen’s d for interpreting the results, it can be concluded that the control group did not achieve a positive impact on body composition transformation. A positive impact was only observed in the variables TMW kg, TTW kg, and LLW kg. The results show (Cohen’s d) that the control group achieved a large impact on body composition transformation in the following variables: TRF % (0.35), TBF % (0.27), TBW % (0.26), RHF % (0.16), RLF % (0.15), LHF % (0.14), and TMW kg (0.14). A moderate impact was observed in the variables LLF % (0.12), TTW kg (0.11), and LLW kg (0.07), while a small impact was observed in the variables LHW kg (0.03) and MIN kg (0.02). Additionally, it is interesting to compare the values of Cohen’s d (Figure 1) for all three groups.

### 3.2. Differences Between Zumba and MoFit Programs’ Effects on Body Composition

In Table 6, the results of the multivariate analysis of covariance for the applied body composition variables between the experimental groups and the control group at the final measurement, with neutralisation of the differences at the initial measurement, are presented. The results show that there is a significant difference at the multivariate level between the experimental groups and the control group after the experimental programs, with a significance level of (F = 11.590; *p* = 0.000).

Based on the value of the Partial Eta Squared (0.79), a large effect of the experimental programs on the differences between the groups in the final measurement can be observed.

Table 7 presents the results of the univariate analysis of covariance for the applied variables assessing body composition between the experimental and control groups at the final measurement, with adjustments for initial measurement differences. The numerical differences between the adjusted mean values (Adj. Means) favour the better results of the experimental groups.

The results indicate that there is a significant difference (*p* < 0.01) at the univariate level for all applied variables: TBF % (F = 37.626; *p* = 0.000), RHF % (F = 20.290; *p* = 0.000), LHF % (F = 26.426; *p* = 0.000), TRF % (F = 11.016; *p* = 0.000), RLF % (F = 28.654; *p* = 0.000), LLF % (F = 11.067; *p* = 0.000), TMW kg (F = 67.794; *p* = 0.000), RHW kg (F = 30.129; *p* = 0.000), LHW kg (F = 19.759; *p* = 0.000), TTW kg (F = 7.810; *p* = 0.001), RLW kg (F = 40.782; *p* = 0.000), LLW kg (F = 43.644; *p* = 0.000), TBW % (F = 34.086; *p* = 0.000), and MIN kg (F = 5.030; *p* = 0.008).

**Figure 2:**
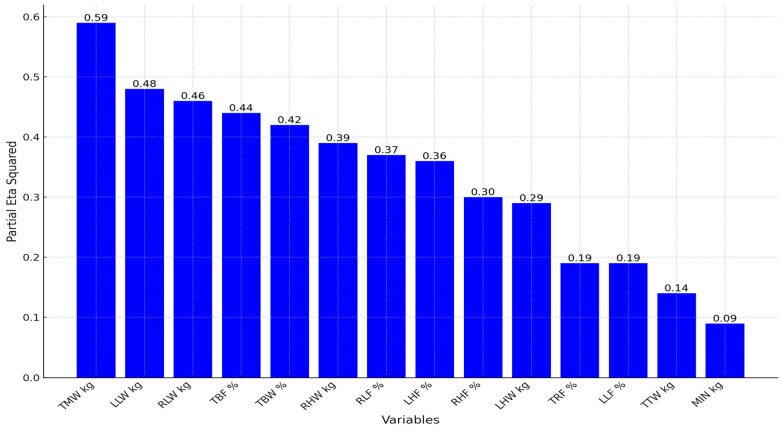
The Partial Eta Squared values for the body composition variables.

Based on the value of the Partial Eta Squared (Figure 2), a large effect of the experimental programs can be observed for all applied variables: TMW kg (0.59), LLW kg (0.48), RLW kg (0.46), TBF % (0.44), TBW % (0.42), RHW kg (0.39), RLF % (0.37), LHF % (0.36), RHF % (0.30), LHW kg (0.29), TRF % (0.19), LLF % (0.19), and TTW kg (0.14). Therefore, it is evident that the Zumba and MoFit experimental programs have achieved significant effects on body composition at the univariate level. A moderate effect was observed only for the variable MIN kg (0.09).

**Figure 3:**
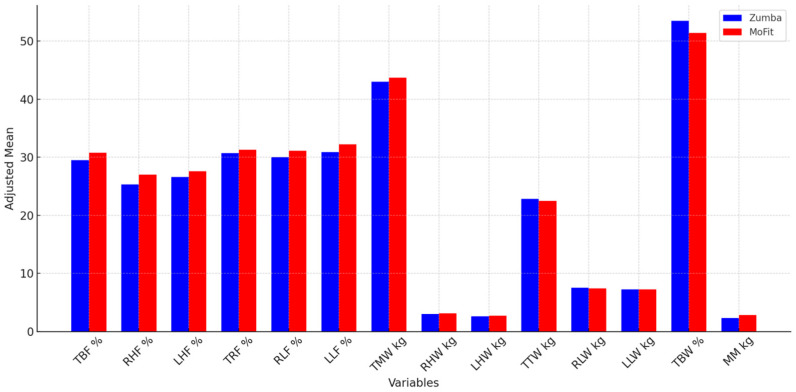
Adjusted mean values of the Zumba and MoFit groups for body composition variables.

When examining the difference in the effects of the two programs (Figure 3), based on the results of the Bonferroni test, it can be observed that the Zumba program achieved significantly greater effects compared to the MoFit group in most of the body composition variables (TBF %, RHF %, LHF %, RLF %, LLW kg, RHW kg, LHW kg, TTW kg, LLW kg, and TBW %), while for the TMW kg variable, better effects were achieved by the participants in the MoFit group. For the variables (TRF %, RLW kg, and MIN kg), no differences were observed between the Zumba and MoFit experimental groups.

## 4. Discussion

The primary aim of this study was to determine the effects of two different fitness programs, Zumba and MoFit, on women’s body composition. The findings confirm that both group fitness programs significantly improve body composition. Moreover, the Zumba program demonstrated a more pronounced positive effect than the MoFit program. As a result, the hypothesis is fully accepted, and the outcomes align with expectations.

The study’s reliability and validity are strongly supported by several factors. First, both initial and final measurements were conducted using standardised, valid, and reliable assessment tools [23,24,25]. Second, all measurements were performed at the same time of day (between 08:00 and 10:00 a.m.) to account for potential daily fluctuations in fitness testing results [27,28,29,30,31]. Future studies could explore variations in body composition testing between morning and evening sessions. Third, sample homogeneity was maintained, with similar numbers of participants in each group (E1: n = 33, E2: n = 31, C: n = 34) and comparable baseline characteristics in terms of height, body mass, and BMI. It is interesting to mention the average age of the participants (E1: 26.5 years, E2: 31.2 years, and C: 25.9 years), with all three groups falling into the category of young adults. This life stage is characterised by a high level of physical activity and metabolic capacity, which is relevant for interpreting the results related to body composition and responses to group fitness programs [9]. Furthermore, the ANCOVA results showed moderate differences in age between the groups (F = 4.997; Partial Eta Squared = 0.09). Finally, the study followed previous research recommendations, applying group fitness sessions 2–3 times per week with a 48 h recovery period between sessions [32,33]. This approach minimises the risk of overtraining and ensures optimal recovery, preventing excessive muscle fatigue that could affect training quality [34].

The Zumba program had a substantial positive impact (*p* < 0.01) across all variables (Cohen’s d: 0.24–0.83), while the MoFit program also showed significant impact (*p* < 0.01; *p* < 0.05) on most variables (Cohen’s d: 0.13–0.89), except for RHW kg and LHW kg. In contrast, the control group exhibited no notable body composition improvements, except for minor positive changes in TMW kg, TTW kg, and LLW kg (*p* < 0.05; Cohen’s d: 0.07–0.14). On the other hand, for the variables TBF %, RHF %, LHF %, TRF %, RLF %, LLF %, and TBW %, the control group showed a deterioration in results (*p* < 0.01, *p* < 0.05; Cohen’s d: 0.12–0.35). Possible causes for these changes may include seasonal effects, dietary changes, stress levels, and physical inactivity, which were beyond our control. Similar outcomes were reported in a study by Wang et al. [35], confirming that group fitness programs lead to significant body composition changes over time.

Furthermore, ANCOVA and MANCOVA analyses demonstrated the significant effects (*p* < 0.01) of Zumba and MoFit programs across all body composition variables, with Partial Eta Squared values (0.14–0.59) confirming large effects. A key finding is that improvements in body composition in both experimental groups were the result of fat reduction, along with the maintenance or increase in muscle mass, total body water, and mineral content, the primary aim of this study. The results align with previous research, indicating that group fitness programs effectively reduce fat, increase muscle mass, and improve overall body composition [36,37,38].

Given the study’s design—including two experimental groups and one control group—it was essential to compare the effectiveness of Zumba and MoFit. The Bonferroni test results revealed that Zumba had significantly greater effects than MoFit in most body composition variables, while no differences were found for TRF %, RLW kg, and MIN kg. These findings align with prior studies, which have shown that a 12-week Zumba program (2× per week) is more effective than general fitness programs for improving body composition [19].

The greater reductions in body fat observed in the Zumba group compared to the MoFit group can be attributed to Zumba’s high-energy expenditure and alternating high- and moderate-intensity intervals. Zumba training promotes fat oxidation and increases basal metabolic rate, resulting in fat tissue reduction [39]. This enhances energy metabolism, increases the efficiency of ATP production, and boosts the utilisation of fat as an energy source, contributing to further fat reduction. Furthermore, the greater effectiveness of Zumba compared to MoFit in reducing body fat and improving body composition parameters may be attributed to a higher excess post-exercise oxygen consumption (EPOC) effect [40] and more complex neuromuscular activation characteristic of choreographed dance programs. The intensity and dynamism of dance movements in Zumba increase the EPOC, thereby contributing to higher total energy expenditure and more efficient fat reduction. Additionally, Zumba involves complex and coordinated full-body movements, including proprioception, balance, and rhythm. These factors collectively contribute to body fat reduction as well as better muscular and cardiovascular system adaptation. Moreover, Zumba training increases insulin sensitivity and optimises glucose regulation, which is crucial for preventing metabolic syndrome and improving overall metabolic health [17,18,19].

On the other hand, the MoFit program demonstrated a superior effect on TMW kg. The inclusion of resistance exercises in MoFit contributed to greater muscle mass gains in this variable. While Zumba is highly effective for fat reduction, its emphasis on high-energy movement does not provide the same stimulus for increasing muscle mass as resistance-based training programs like MoFit.

In addition to their positive impact on fat reduction and the preservation and increase in muscle mass, both Zumba and MoFit programs demonstrated beneficial effects on TBW % and MIN kg, confirming the significance of both exercise interventions. However, Zumba exhibited greater effects compared to MoFit, which may be attributed to the dynamic nature of the activity, as well as the more extensive involvement of the whole body during choreographed dance routines. Specifically, the dynamic and rhythmic movements of Zumba contributed to improved hydration adaptation [41]. On the other hand, MoFit was of moderate intensity, which may explain the slightly reduced effect on TBW % [42].

Furthermore, the results showed that both experimental groups achieved significant effects compared to the control group regarding the MIN kg variable, although no differences were observed between the experimental groups. This indicates that both Zumba and MoFit stimulate osteogenesis and mineral metabolism, which is reflected in the increased MIN kg values. These findings are highly relevant for several reasons. Minerals are essential for maintaining bone density, preventing osteopenia and osteoporosis, supporting skeletal stability, particularly in women, and regulating muscle contraction, nerve conduction, and electrolyte balance [43]. On the other hand, TBW % represents a crucial hydration parameter, and improvements in this variable following both programs indicate enhanced cellular function and metabolic efficiency [44].

### Strengths, Limitations, Future Directions, and Practical Applications

The strength of this study lies in being the first to comprehensively examine body composition parameters under the influence of group fitness programs Zumba and MoFit. Furthermore, the MoFit program has been validated in this way and presented as an innovative and modern approach to group exercise that contributes to improving body composition in women. Additionally, this is one of the first studies to apply the Zumba program over a 10-week period. Previous studies have most commonly applied this program in women for durations of 8 or 12 weeks [19,21,22,32,34,43,45,46].

Despite the study’s robust methodology, certain limitations must be acknowledged. Factors such as the menstrual cycle, contraceptive use, and endocrine conditions were not considered in this study, which may have influenced the results related to body composition [47,48]. It is recommended that future research include these factors for a more precise analysis. The experimental programs did not account for participants’ dietary habits, which can significantly influence improvements in body composition [10,49]. However, although the authors of this study did not conduct a formal analysis of caloric intake, macronutrients, or supplementation, the participants were informed about the principles of proper nutrition at the beginning and throughout the course of the program. They were also given advice on balanced nutrition, with an emphasis on adequate intake of protein, carbohydrates, and fats in accordance with training objectives and the increased energy demands during the study period. Therefore, future studies should incorporate nutritional tracking (for example, Food Diaries, Food Frequency Questionnaires, and Cronometers), as well as additional factors such as sleep, stress, and recovery, which may also impact results. Additionally, a recommendation for future research would be to use the RPE scale and other measures of internal load, which would contribute to greater accuracy and validity of the results. Finally, this study did not take into account the participants’ ages; this study included those participants who fully completed the experimental treatments and focused on being within the young adult age category.

From a practical perspective, this research underscores the importance of incorporating group fitness programs into daily life and provides recommendations for targeted programs aimed at improving body composition—fat reduction, lean mass increase, total body water, mineral content, as well as muscle preservation and increase—all of which are key factors in maintaining a healthy lifestyle. Fitness professionals can utilise these results to more precisely tailor exercise programs according to individual objectives and needs. Additionally, they can integrate elements from both programs to create effective and personalised workouts that promote optimal improvements in body composition and overall health in women.

From a scientific perspective, the results provide a basis for further research in the field of group fitness programs, particularly regarding training duration and intensity, as well as their effects on other physical fitness parameters (cardiorespiratory fitness, flexibility, and motor fitness) and on different population groups. Additionally, the findings may serve as a starting point for the development of new, interdisciplinary fitness programs and the evaluation of their effects.

## 5. Conclusions

The main findings of this study confirmed the positive effects of both Zumba and MoFit on body composition. The improvement in body composition in the experimental groups was achieved through a reduction in body fat, preservation or increase in muscle mass, and an increase in total body water and mineral content. When comparing the effects of the two programs, the results showed that the Zumba program had significantly greater effects than the MoFit program on most body composition variables. The results obtained from the longitudinal study provide both a scientific-theoretical and practical contribution to the field, offering a precise answer regarding the effects of the designed experimental programs on women’s body composition. More specifically, fitness trainers and experts require efficient group exercise programs that will contribute to improving body composition in a short period of time. Therefore, we can conclude that the Zumba and MoFit programs are effective in reducing body fat, increasing muscle mass, total body water, and mineral content, whereas the control group did not achieve positive changes.

## Figures and Tables

**Table 1:** Measuring instruments for assessing body composition.

No.	Variable	Measurement	Abbreviation
1.	Total body fat	%	TBF %
2.	Right arm fat	%	RHF %
3.	Left arm fat	%	LHF %
4.	Trunk fat	%	TRF %
5.	Right leg fat	%	RLF %
6.	Left leg fat	%	LLF %
7.	Total muscle weight	kg	TMW kg
8.	Total weight of right arm	kg	RHW kg
9.	Total weight of left arm	kg	LHW kg
10.	Total weight of trunk	kg	TTW kg
11.	Total weight of right leg	kg	RLW kg
12.	Total weight of left leg	kg	LLW kg
13.	Total body water	%	TBW %
14.	Minerals	kg	MIN kg

**Table 2:** Basic information about the experimental Zumba and MoFit programs.

Groups	E1 Group Zumba	E2 Group MoFit	Control Group
Frequency	3× times per week	3× times per week	- - - -
Duration of the training	65–70 min	55–60 min	- - - -
Duration of the program	10 weeks	10 weeks	- - - -
Number of trainings	30×	30×	- - - -
Intensity	75–85% HR_max_	60–75% HR_max_	- - - -

**Table 3:** Differences in body composition between the initial and final measurements of the Zumba experimental group.

Variable	Mean I	Mean F	Mean Diff	SD	t	*p*	Cohen’s d
TBF %	32.46	29.71	2.74	2.34	6.735	0.000 **	0.58 ***
RHF %	29.79	27.20	2.59	2.38	6.254	0.000 **	0.55 ***
LHF %	30.74	28.09	2.64	2.41	6.312	0.000 **	0.55 ***
TRF %	31.79	29.98	1.81	3.11	3.335	0.000 **	0.25 ***
RLF %	33.17	30.72	2.45	2.24	6.287	0.000 **	0.55 ***
LLF %	34.50	32.15	2.35	2.97	4.552	0.000 **	0.39 ***
TMW kg	43.59	44.70	−1.11	0.55	−11.526	0.000 **	0.80 ***
RHW kg	2.21	2.37	−0.15	0.09	−9.460	0.000 **	0.73 ***
LHW kg	2.23	2.37	−0.13	0.09	−8.023	0.000 **	0.66 ***
TTW kg	24.08	24.50	−0.41	0.39	−6.158	0.000 **	0.54 ***
RLW kg	7.67	7.85	−0.17	0.09	−11.078	0.000 **	0.79 ***
LLW kg	7.42	7.65	−0.23	0.11	−12.862	0.000 **	0.83 ***
TBW %	50.08	53.38	−3.30	3.04	−6.233	0.000 **	0.55 ***
MIN kg	2.38	2.40	−0.02	0.04	−3.200	0.003 **	0.24 ***

Legend: Mean I—mean at the initial measurement; Mean F—mean at the final measurement; Mean Diff—difference between the means of the initial and final measurements; SD—standard deviation; t—result of the *t*-test; *p*—significance level ** < 0.01; Cohen’s d—Cohen’s effect size = 0.01 (small impact); ** ≥ 0.06 (moderate impact); *** ≥ 0.14 (large impact).

**Table 4:** Differences in body composition between the initial and final measurements of the MoFit experimental group.

Variable	Mean I	Mean F	Mean Diff	SD	t	*p*	Cohen’s d
TBF %	33.68	32.07	1.60	1.37	6.503	0.000 **	0.58 ***
RHF %	29.22	28.11	1.11	1.72	3.610	0.001 **	0.30 ***
LHF %	30.14	28.81	1.32	1.24	5.94	0.000 **	0.80 ***
TRF %	34.27	32.77	1.49	1.37	6.053	0.000 **	0.53 ***
RLF %	34.32	33.14	1.17	1.00	6.503	0.000 **	0.58 ***
LLF %	35.36	34.05	1.31	1.42	5.098	0.000 **	0.46 ***
TMW kg	40.10	41.59	−1.48	0.52	−15.634	0.000 **	0.89 ***
RHW kg	2.12	2.12	0.00	0.09	−0.183	0.856	0.01 *
LHW kg	2.13	2.15	−0.2	0.09	−1.076	0.290	0.03 *
TTW kg	21.48	21.64	−0.16	0.42	−2.138	0.041 *	0.13 **
RLW kg	7.31	7.46	−0.14	0.08	−10.185	0.000 **	0.77 ***
LLW kg	6.99	7.14	−0.15	0.08	−9.704	0.000 **	0.75 ***
TBW %	48.01	49.50	−1.49	1.35	−6.121	0.000 **	0.55 ***
MIN kg	2.20	2.22	−0.02	0.04	−3.503	0.001 **	0.29 ***

Legend: Mean I—mean at the initial measurement; Mean F—mean at the final measurement; Mean Diff—difference between the means of the initial and final measurements; SD—standard deviation; t—result of the *t*-test; *p*—significance level * < 0.05, ** < 0.01; Cohen’s d—Cohen’s effect size = * ≥ 0.01 (small impact); ** ≥ 0.06 (moderate impact); *** ≥ 0.14 (large impact).

**Table 5:** Differences in body composition between the initial and final measurements of the control group.

Variable	Mean I	Mean F	Mean Diff	SD	t	*p*	Cohen’s d
TBF %	30.86	31.64	−0.78	1.22	−3.745	0.001 **	0.27 ***
RHF %	24.68	24.88	−0.2	0.47	−2.518	0.017 *	0.16 ***
LHF %	26.00	26.24	−0.23	0.59	−2.343	0.025 *	0.14 ***
TRF %	32.09	32.37	−0.28	0.38	−4.313	0.000 **	0.35 ***
RLF %	29.55	29.78	−0.22	0.54	−2.469	0.019 *	0.15 ***
LLF %	30.23	30.40	−0.16	0.43	−2.249	0.031 *	0.12 **
TMW kg	40.83	40.98	−0.14	0.35	−2.425	0.021 *	0.14 ***
RHW kg	2.14	2.15	−0.01	0.07	−0.442	0.661	0
LHW kg	2.12	2.13	−0.01	0.07	−1.094	0.282	0.03 *
TTW kg	21.67	21.75	−0.07	0.21	−2.020	0.052	0.11 **
RLW kg	7.42	7.42	0.00	0.08	0	1.000	0
LLW kg	7.32	7.35	−0.02	0.08	−1.605	0.118	0.07 **
TBW %	51.39	50.75	0.63	1.07	3.450	0.002 **	0.26 ***
MIN kg	2.15	2.15	−0.00	0.01	−1.000	0.325	0.02 *

Legend: Mean I—mean at the initial measurement; Mean F—mean at the final measurement; Mean Diff—difference between the means of the initial and final measurements; SD—standard deviation; t—result of the *t*-test; *p*—significance level * < 0.05, ** < 0.01; Cohen’s d—Cohen’s effect size = * ≥ 0.01 (small impact); ** ≥ 0.06 (moderate impact); *** ≥ 0.14 (large impact).

**Table 6:** Multivariate analysis of covariance of experimental groups and control group for body composition.

Wilks’ Lambda	F	df1	df2	*p*	Partial Eta Squared
0.087	11.590	210	235	0.000 **	0.79 ***

Legend: Wilk’s Lambda—Wilks’ Lambda test; F—F approximation; df1, df2—degrees of freedom; *p*—significance of differences ** < 0.01; Partial Eta Squared—effect size, ** ≥ 0.06 (moderate effect), *** ≥ 0.14 (large effect).

**Table 7:** Univariate analysis of covariance of experimental groups and control group for body composition.

Variable	Adj. Mean E1	Adj. Mean E2	Adj. Mean C	F	*p*	Bonferroni	Partial Eta Squared
TBF %	29.56	30.78	32.97	37.626	0.000 **	E1 > C **; E1 > E2 **; E2 > C **	0.44 ***
RHF %	25.44	26.86	27.72	20.290	0.000 **	E1 > C **; E1 > E2 **	0.30 ***
LHF %	26.45	27.71	28.84	26.426	0.000 **	E1 > C **; E1 > E2 **; E2 > C **	0.36 ***
TRF %	30.84	31.24	32.94	11.016	0.000 **	E1 > C **; E2 > C **	0.19 ***
RLF %	29.91	31.30	32.25	28.654	0.000 **	E1 > C **; E1 > E2 **; E2 > C *	0.37 ***
LLF %	31.04	32.17	33.19	11.067	0.000 **	E1 > C **; E1 > E2 *	0.19 ***
TMW kg	42.66	43.00	41.67	67.794	0.000 **	E1 > C **; E2 > C **; E2 > E1 *	0.59 ***
RHW kg	2.31	2.16	2.16	30.129	0.000 **	E1 > C **; E1 > E2 **	0.39 ***
LHW kg	2.30	2.18	2.17	19.759	0.000 **	E1 > C **; E1 > E2 **	0.29 ***
TTW kg	22.83	22.59	22.50	7.810	0.001 **	E1 > C **; E1 > E2 *; E2 > C *	0.14 ***
RLW kg	7.65	7.62	7.47	40.782	0.000 **	E1 > C **; E2 > C **	0.46 ***
LLW kg	7.49	7.40	7.27	43.644	0.000 **	E1 > C **; E1 > E2 **; E2 > C **	0.48 ***
TBW %	53.21	51.13	49.44	34.086	0.000 **	E1 > C **; E1 > E2 **; E2 > C **	0.42 ***
MIN kg	2.27	2.27	2.24	5.030	0.008 **	E1 > C *; E2 > C *	0.09 **

Legend: Adj. Mean—Adjusted mean values; F—F-test coefficient value; *p*—significance level ** < 0.01; Bonferroni—summary of Bonferroni test results; Partial Eta Squared—effect size * ≥ 0.01 (small effect), ** ≥ 0.06 (moderate effect), *** ≥ 0.14 (large effect).

## Data Availability

The authors will provide the raw data supporting the conclusions of this article upon request.
